# Activity and Function in Human Cells of the Evolutionary Conserved Exonuclease Polynucleotide Phosphorylase

**DOI:** 10.3390/ijms23031652

**Published:** 2022-01-31

**Authors:** Federica A. Falchi, Roberto Pizzoccheri, Federica Briani

**Affiliations:** Dipartimento di Bioscienze, Università degli Studi di Milano, 20133 Milano, Italy; federica.falchi@unimi.it (F.A.F.); roberto.pizzoccheri@unimi.it (R.P.)

**Keywords:** polyribonucleotide phosphorylase, PNPase, *PNPT1*, RNA decay, RNA stability, RNA binding protein, exoribonuclease, mitochondrial RNA, RNA degradosome

## Abstract

Polynucleotide phosphorylase (PNPase) is a phosphorolytic RNA exonuclease highly conserved throughout evolution. Human PNPase (hPNPase) is located in mitochondria and is essential for mitochondrial function and homeostasis. Not surprisingly, mutations in the *PNPT1* gene, encoding hPNPase, cause serious diseases. hPNPase has been implicated in a plethora of processes taking place in different cell compartments and involving other proteins, some of which physically interact with hPNPase. This paper reviews hPNPase RNA binding and catalytic activity in relation with the protein structure and in comparison, with the activity of bacterial PNPases. The functions ascribed to hPNPase in different cell compartments are discussed, highlighting the gaps that still need to be filled to understand the physiological role of this ancient protein in human cells.

## 1. Introduction

Polynucleotide phosphorylase (PNPase) is a phosphorolytic exoribonuclease that degrades the RNA from the 3′-end to the 5′-end generating nucleoside diphosphates (NDPs). PNPase has been widely conserved throughout evolution and orthologues are present not only in distantly related bacterial groups, but also in protists, metazoa and green plants. Conversely, PNPase is absent in some unicellular Eukarya, among which the model organism *Saccharomyces cerevisiae*, and in Archaea [1,2,3]. 

The research on bacterial PNPase started more than 60 years ago in Severo Ochoa’s laboratory [4], but although a PNPase-like activity in humans and rats was reported early [5,6], studies on the human orthologous protein began much later, when the human PNPase (hPNPase) cDNA was firstly cloned [7]. Later on, hPNPase protein was demonstrated to be located in mitochondria [8]. Recently, mutated variants of hPNPase have been associated to different pathologies, mostly neurological, with a wide spectrum of symptoms and severity [9,10,11].

Here we review hPNPase activity, functions and interactions with RNAs and other proteins. A comprehensive overview of the pathologies that have been associated to hPNPase mutations is out of the scope of this review and we will refer to this aspect limitedly as to what we can infer about the physiological hPNPase function and biochemical activity from studies on the pathological hPNPase variants.

## 2. Organization and Regulation of the *PNPT1* Gene

### 2.1. PNPT1 Gene and mRNA Features

In 2002, a cDNA encoding the human PNPase (hPNPase) was cloned in P.B. Fisher laboratory from interferon β (INF-β)-treated melanoma HO-1 cells in a screening campaign aimed to identify genes upregulated in both senescence and terminal differentiation. The gene was named *old-35* (hereafter referred-to as *PNPT1* according to the current nomenclature) and found to encode a protein of 783 amino acids with high similarity to bacterial PNPase [7]. 

The *PNPT1* gene is located on chromosome 2 and spans *ca*. 59 kb in the 2p15-2p16.1 region (Ensemble gene ID: ENSG00000138035; coordinates 55,634,061-55,693,863, minus strand), with its coding region divided among 28 exons [12]. Two pseudogenes are present at locations 3p26.2 and 7q31.32. *PNPT1* belongs to the group of human core fitness genes [13] and its murine orthologue is essential in mouse embryonic fibroblasts (MEF) [14]. Conversely, MEFs conditioned to grow in uridine-supplemented medium tolerate a *PNPT1* mutation causing hPNPase loss. These cells are respiration-deficient and devoid of mitochondrial DNA (mtDNA) and rely on increased glycolysis for their energetic metabolism [15]. Indeed, hPNPase under-expression results in strong alteration of mitochondrial function and morphology, loss of mtDNA and decreased respiratory chain activity in both human and mouse cells, highlighting the relevance of the protein in mammalian mitochondrial homeostasis. Accordingly, *Pnpt1^KO/KO^* mice, with the complete deletion of exon 2 that in turn causes frame-shifting, are embryonic lethal at day 8 [14,15,16]. 

The main *PNPT1* transcript according to Matched Annotation from NCBI and EBI-EMBL (MANE Select) is 4549 nt long, with 5’- and 3’-UTRs 21 nt and 2176 nt long, respectively. Two *PNPT1* transcripts estimated as about 2.6 and 4.3 kb long were originally identified in HO-1 melanoma cells, with the shorter one being more abundant than the long one. Both mRNAs contained the complete hPNPase coding region and differed for the length of the 3’- UTR, suggesting that they can derive from polyadenylation at different sites [7]. A similar transcription pattern was found in various human tissues [17], suggesting that the primary *PNPT1* transcript is not subject to significant alternative splicing, at least in the tested tissues/conditions.

### 2.2. PNPT1 Regulation and Expression in Human Tissues

*PNPT1* was found to be an interferon I early response gene, as its transcription was induced by IFN-β, and to a lesser extent by IFN-α, in different cancer cell lines and skin fibroblasts [7,12]. To our knowledge, regulation of *PNPT1* transcription by other stimuli has not been reported so far, although binding sites for various transcription factors not related to interferon response are present in the *PNPT1* promoter region [18]. Given that *PNPT1* seems essential for cell survival [14,15,16], it is likely that all cells have a certain level of hPNPase. Accordingly, *PNPT1* expression in the absence of IFN-β stimulation has been found in different types of cells [14,18,19]. 

The *PNPT1* promoter lacks recognizable TATA and CAAT elements, whereas it encompasses a GC-rich Sp1 site, a Gamma Activated Sequence (GAS) flanked by two Interferon Regulatory Factor-1 (IRF-1) binding sites and an IFN Stimulated Regulatory Element (ISRE) [7,12]. The ISRE (GAAAAN(N)GAAA sequence in the *PNPT1* promoter) plays a major role in *PNPT1* expression regulation, as point mutations within ISRE abolish IFN-dependent *PNPT1* transcription induction [12].

**Figure 1:**
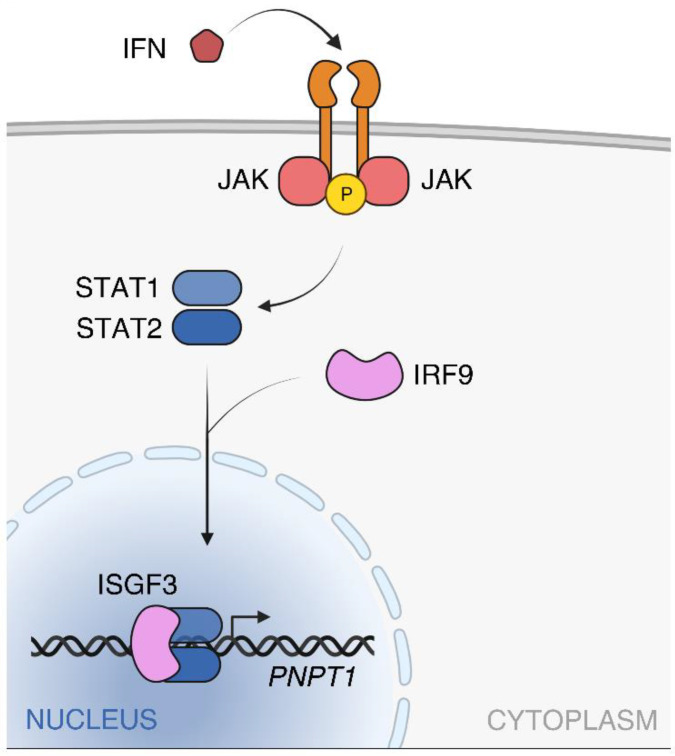
*PNPT1* transcription induction by type I interferon. IFN-β and INF-α (IFN) induce the JAK-STAT signaling cascade described in the text leading to *PNPT1* transcription activation by ISGF3. STAT2/IRF9 complex, which could also bind the ISRE, is not shown. Created with Biorender.com. Available online: https://biorender.com (accessed on 27 January 2022).

The ISRE is responsive to the ISGF3 (IFN-Stimulated Gene Factor 3) and STAT2/IRF9 (Signal Transducer and Activator of Transcription 2-IFN Regulatory Factor 9) complexes, which are formed upon JAK (Janus kinase)-STAT signaling cascade activation in response to IFNα and INFβ (type I IFN) or IFNλ (type III IFN) [20,21]. JAK-dependent phosphorylation of STAT1 and STAT2 leads to homo- and heterodimerization of the two phosphorylated proteins. STAT2 homodimers and STAT1-STAT2 heterodimers interact with IRF9 to form STAT2/IRF9 and ISGF3 complexes, respectively, that enter the nucleus and bind to ISRE regions activating the transcription of hundreds of interferon sensitive genes (ISGs), among which *PNPT1* (Figure 1).

In principle, STAT1 homodimerization and IRF-1 expression could be induced by type I and II IFNs and STAT1 homodimers (i.e., the GAF factor) could bind the GAS region, which is also present in the *PNPT1* promoter together with IRF-1 binding sites [21]. However, the deletion of IRF-1 or GAS sites does not impair IFN-β-dependent *PNPT1* induction suggesting that these sites have a minor role with respect to the ISRE in *PNPT1* regulation; in addition, minimal responsiveness of *PNPT1* to the type II interferon IFN-γ was found [12]. Accordingly, IRF-1 seems to have an accessory role in the regulation of ISGs [22].

Regulation of the *E. coli pnp* gene, encoding EcPNPase, mainly occurs at post-transcriptional level through a complex mechanism involving the 5’-UTR of the *pnp* mRNA, the endonuclease RNase III and EcPNPase itself, which negatively autoregulates its own expression by repressing translation and causing mRNA destabilization [2,23,24,25]. The stability of IFN early response mRNAs is also modulated post-transcriptionally by *cis*-acting adenylate–uridylate-rich element (ARE) located in their 3’-UTRs [26,27,28]. Three AUUUA sequences are present in the *PNPT1* 3’-UTR; however, in HO-1 cells, *PNPT1* mRNA half-life (estimated as equal to 6h) is not altered in presence or absence of IFN-β [12], suggesting that mRNA stability regulation has a minor role, if any, in the INF-dependent induction of *PNPT1*, at least in this cell type.

According to bulk tissue gene expression analysis reported in Genotype Tissue Expression databank (GTEx Portal), and consistent with an essential role of the protein, *PNPT1* is ubiquitously expressed in human tissues with the lowest expression level in whole blood and the highest in cerebellar hemisphere and in cultured fibroblasts and EBV-transformed lymphocytes. hPNPase is also widely expressed in human cell lines including HO-1, WM238 and SK-MEL 110/470 melanoma cell lines, breast carcinoma, osteosarcoma and Hela cells [7]. Expression of PNPase in different tissues (i.e., brain, hearth, liver, lung, inner ear and kidney) has been reported also in *Mus musculus* [29]. Conversely, in *Danio rerio* (i.e., zebrafish) the expression pattern of the orthologous protein changes during development, being ubiquitous immediately after fertilization and becoming more and more restricted and limited to the tectum, the gill arches, and the developing ear at 48 h and 120 h post-fertilization [29]. Interestingly, in human, hearing loss is frequently associated with *PNPT1* mutations, with or without other neurological symptoms depending on the mutations, and *PNPT1* knock-out in inner ear hair cells causes hearing loss in mice [10,15,29]. 

## 3. Structure of hPNPase: A Catalytic Core Capped by an RNA Binding Pore

### 3.1. Protomer Domains and Quaternary Structure of hPNPase

**Figure 2:**
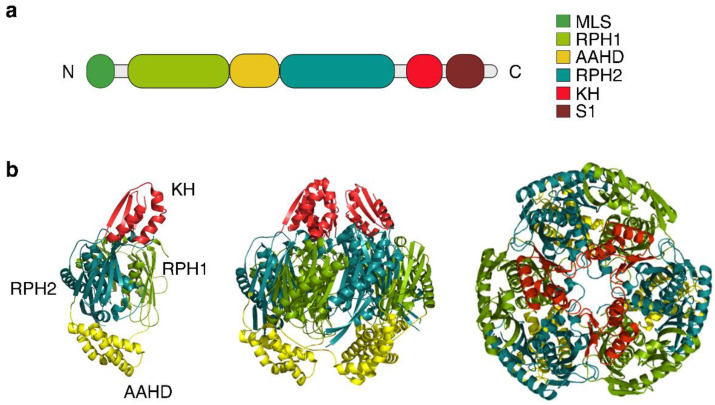
Structure of hPNPase. (**a**) Domain organization. The boxes represent the five evolutionarily conserved motifs that include MLS, mitochondrial localization sequence; AAHD, All α-helical domain; RPH1, Ribonuclease PH domain 1; RPH2, Ribonuclease PH domain 2; KH, K homology domain; S1, S1 domain. N, N-terminus; C, C-terminus. Created with Biorender.com. Available online: https://biorender.com/ (accessed on 27 January 2022). (**b**) Structure of monomeric (left) and trimeric (central, side view, and right, top view) truncated (devoid of S1 domain) hPNPase (PDB ID: 3U1K). The domains are colored as in panel a.

PNPases are members of the PDX family of exoribonucleases [30] and in all organisms share a similar domain structure, consisting of two RNase PH like domains (RPH1 and RPH2), separated by an all-α-helical domain (AAHD), and followed by the KH and S1 RNA binding domains [31]. An N-terminal (N-ter) mitochondrial localization sequence is also present in the human enzyme (MLS, aa 1-45; Figure 2a). No physiological isoforms of hPNPase deviating from this general organization have been described so far, in agreement with the lack of alternatively spliced forms of the *PNPT1* mRNA.

The crystal structures of *Streptomyces antibioticus*, *E. coli*, *Caulobacter crescentus*, *Homo sapiens* PNPases reveal a characteristic trimeric architecture with the six RNase PH domains of the three protomers assembled in a ring-like conformation delimiting a central channel where the active site is located [32,33,34,35,36] (Figure 2b).

In hPNPase, the three KH domains form a pore capping the catalytic core. Each KH domain contacts not only the other two KH domains, but also the RPH1 domain of the adjacent monomer, suggesting that the KH domain contributes also to the formation and stabilization of the trimer [35]. Contribution of the RNA binding domains to the overall stability of the trimeric enzyme has been demonstrated also for EcPNPase [36,37,38,39]. Less clear is the positioning of the S1 domain because the structure of an S1-truncated hPNPase, and not of the full-length enzyme, was resolved [35]. Moreover, whenever present in crystals of bacterial enzymes, this part of PNPase is disordered, suggesting that it may have high mobility [32,33,36,40]. The recent cryo-EM structure of the full-length EcPNPase confirmed that the KH-S1 RNA binding module has high mobility in the absence of the RNA substrate. When the enzyme is in the phosphorolytic mode (i.e., binding the RNA substrate), the S1 and the KH domains reposition around the captured RNA guiding it towards the core channel [41].

It has been suggested that hPNPase may have a narrower central channel with respect to bacterial orthologues [35], but the comparison is not straightforward because PNPase versions with or without the KH and/or the S1 domains were analyzed and it is known that the lack of the RNA binding domains may affect the packing of the enzyme [36]. Moreover, the diameter of at least some regions of the central channel seems to be variable depending on the presence or absence of bound RNA [32,33,34,42], suggesting that different conformations of the enzyme core may exist.

Notably, PNPase architecture resembles that of exosomes, protein complexes involved in RNA processing and degradation in Archaea and Eukarya [3,43]. Like in PNPase, in the exosome six RNase PH-like proteins form a ring-like structure and three additional S1/KH-containing proteins form a pore on the top of the ring [44]. However, while archaeal exosome is a phosphorolytic machine-like PNPase [45], eukaryotic exosomes (except in plants [46]) are hydrolytic machines, generating NMPs from RNA digestion due to the association of hydrolytic exoribonuclease subunits to the catalytically inactive RPH core [47,48].

### 3.2. Post-Translational Modifications and Interaction with Small Regulatory Molecules

Dataset obtained from several mass spectrometry (MS)-based proteomics studies indicate that 95 post-translational modification (PTM) sites could be present in hPNPase, most frequently for phosphorylation, acetylation, and ubiquitylation (see PhosphositePlus^®^ database) [49]. However, up to now, only phosphorylation at Ser-776 (discussed in Section 5.3 below) has been experimentally demonstrated and reported to regulate cellular processes [50].

Nothing is known about in vivo modulation of hPNPase activity by small molecules. In the case of bacterial PNPases, modified nucleotides like cyclic-di-GMP and (p)ppGpp have been shown to regulate the activity of the enzyme, at least in vitro [51,52,53]. ATP is also an allosteric inhibitor of EcPNPase and in vitro it impairs both its phosphorolytic and polymerization activities [54], suggesting that EcPNPase may have a major role as an RNA processing enzyme upon metabolic stresses affecting ATP production. To our knowledge, modulation of hPNPase in vitro activity by none of these nucleotides has been analyzed so far.

Another link between the enzyme activity and the central metabolism was established when citrate, an intermediate of the TCA cycle, was shown to bind and regulate the activity of EcPNPase in vitro and in bacterial cells [55]. Interestingly, citrate is also able to down-regulate hPNPase degradative activity in vitro [56]. The concentration of citrate in mitochondria is subject to fluctuations depending on the cellular metabolic state and efficiency of oxidative phosphorylation, and indeed citrate modulates the activity of different enzymes connected with glycolysis, gluconeogenesis, and fatty acid metabolism [57]. Thus, although experimental evidence is still lacking, in theory, hPNPase may be modulated also in vivo, in human cells, by changes in citrate concentrations.

## 4. hPNPase Catalytic and RNA Binding Activity

### 4.1. Catalytic Activity and Active Site Composition

hPNPase is 40% identical and 57% similar [33,58] to EcPNPase, whose enzymatic activity has been studied in deep by different groups over the years. Studies performed on hPNPase, although less exhaustive, have found many similarities, but also relevant differences between the two enzymes with respect to the catalytic activity and the preferred RNA substrates.

In vitro, in presence of Mg^2+^, both EcPNPase and hPNPase can perform RNA phosphorolysis from 3′- to 5′-end, using inorganic phosphate (Pi) to attack the phosphodiester bonds, and the reverse NDP polymerization reaction, which can form heteropolymeric tails at the RNA 3′-end [2,59].

Single-stranded RNAs are processively digested by PNPase producing NDPs and oligoribonucleotides estimated around 4 and 2 nt long for human and *E. coli* PNPase, respectively, that are not further degraded [35,60]. In vitro, hPNPase does not degrade RNAs with 3′-end single-stranded tails shorter than 12 nt long. Similarly, EcPNPase stops degrading RNAs once the single-stranded tail has been shortened to 6–9 nt [35,61,62]. This conserved feature of PNPases has been related to the size of the channel where the active site is located, which can accommodate only single-stranded RNAs, and to the distance between the RNA binding domains and the active site [32,35]. Indeed, efficient and processive degradation relies on the concomitant interaction of the RNA substrate with the RNA binding domains and the catalytic site, a trait that has also been associated with processive RNA hydrolyzing machines [63].

It was reported that hPNPase has 0.1 mM optimal Pi concentration and it is inhibited at Pi concentrations greater than 2.5 mM, which is optimal for the bacterial enzyme [59]. However, phosphorolytic activity was observed in assays performed with hPNPase or the hPNPase-SUPV3L1 complex in presence of 5–7 mM Pi [64,65]. Accordingly, hPNPase-dependent degradation has been shown to occur in the mitochondrial matrix and in the cytosol of mammalian cells, two compartments in which phosphate concentration has been variably estimated between 2 and 10 mM, similar to the concentration found in *E. coli* [66,67,68,69].

Mutational and biochemical studies of hPNPase position the active site in the RPH2 domain, with residues Arg446, Ser484, Asp538, and Asp544 being critical for the catalytic activity of the enzyme [35,59]. The four residues are conserved also in EcPNPase (i.e., Arg399, Ser439, Asp486, and Asp495), whose active site has similar geometry [33,35,36], and missense mutations changing each of them (with the exception of Ser439, for which to our knowledge mutagenesis studies have not been conducted) impair the enzyme catalytic activity [39,59]. According to structural studies performed on different bacterial PNPases, Ser439, together with Ser438 (corresponding to Ser483 in hPNPase), should be involved in phosphate coordination, whereas Asp486, Asp492, and Lys494 (Lys546 in hPNPase) in coordinating the Mg^2+^ ion implicated in the catalytic mechanism [32,33,70]. Although not directly involved in the catalysis, the RPH1 domain participates in the formation of the active site in bacterial PNPase [32,39,42,71] and it seems to be relevant for hPNPase catalysis too, as suggested by defects of pathological hPNPase variants with amino acid changes in the RPH1 domain [72,73].

In vitro and presence of Mn^2+^, the bacterial PNPases can catalyze both DNA phosphorolysis and synthesis of DNA from dNDPs [74,75,76,77]. Consistent with this activity, PNPases of both Gram-negative and Gram-positive bacteria have been implicated in DNA recombination, repair, and mutagenesis [77,78,79,80]. Whether DNA and dNDPs may be substrates also for hPNPase and, if so, what the in vivo relevance of such activity may be on the maintenance of mtDNA are unexplored issues.

### 4.2. Both the Enzyme Core and the KH-S1 Domains Contribute to RNA Binding

Human PNPase prefers to bind poly(U) and poly(G) RNAs, whereas the bacterial enzyme, albeit having low specificity in ssRNA sequence recognition [81], preferentially binds poly(U) and poly(A) RNAs [59,82]. However, the in vivo relevance of the low binding affinity to poly(A) RNA of hPNPase is uncertain, considering that hPNPase has been involved in the regulation of the length of polyadenylated tails of mitochondrial RNAs and the massive degradation of cytosolic polyadenylated mRNAs during apoptosis [64,65].

In *E. coli*, the lack of either (or both) the S1 or the KH domains severely impairs RNA binding, but it does not abolish PNPase-dependent degradation of single-stranded substrates, implying that they can still interact with the enzyme [37,71,83]. Consistent with these observations, residues in the RPH1 domain, and in particular in the FFRR loop, have been implicated in the interaction with the RNA substrate [33,40,42]. The FFRR loop is conserved in PNPases from different organisms with an FX(R/K)RE consensus sequence, where X is any amino acid [42]. The first phenylalanine of the FFRR loop (Phe77) can make a stacking interaction with RNA in both *E. coli* and *C. crescentus* orthologs, which is relevant for the correct positioning of the RNA substrate in the central channel [33,34,41]. In hPNPase, the phenylalanine is replaced by a tyrosine (Tyr116), which can maintain stacking. The second arginine in the EcPNPase FFRR loop (i.e., Arg80) does not bind directly to the RNA, but it establishes long-range interactions ultimately affecting the conformation of the catalytic site via hydrogen bonding with Ser438 in the active site, mediated by Tyr380. This connection seems important to maintain the proper reciprocal orientation between the active site and the RNA substrate [39,42]. Although the region of the FFRR loop is not resolved in the hPNPase structure (PDB-codes 3U1K and 5ZF6), probably because it is highly dynamic in the absence of bound RNA [33,40,42], all residues involved in the hydrogen bonding interaction network described above are conserved also in hPNPase (i.e., Arg119, Tyr427, and Ser438). Mutagenesis studies should be conducted to assess whether these residues are involved in the interaction with the RNA substrate and catalysis.

The KH pore has a major role in RNA binding by hPNPase and a single missense mutation, namely Gly622Asp, abolishes almost completely RNA binding and, in turn, affects the RNA degradation activity [35]. Gly622 is located in the GXXG motif, which is conserved in KH domains found in different proteins and has a major role in nucleic acid binding [84]. The GXXG motif is highly conserved in PNPases of various species and it is located in a β-turn region directly interacting with the RNA substrate [35]. Conversely, it was reported that the S1 domain does not have critical importance for ssRNA binding by hPNPase. Indeed, ΔS1 hPNPase binds RNA almost as tightly and cleaves poly(A) and poly(U) RNA almost as efficiently as the full-length protein [35]. These observations do not rule out that the S1 domain may have a relevant role in the degradation of natural RNA substrates of hPNPase. *PNPT1* alleles encoding hPNPase variants with missense mutations in the S1 domain have been found in patients with serious neurological symptoms in mixed heterozygosis with alleles encoding variants mutated either in a different hPNPase domain or, in one case, at a different position in the S1 domain [9,10,73]. Thus, a relatively modest defect detected in in vitro tests can have grave consequences in vivo. This may reflect on the one hand the limitations of the in vitro assays, in which a small repertoire of model RNAs is usually analyzed, and on the other hand the multifaceted cellular role of hPNPase that is not restricted to RNA degradation (discussed in the following Section 5).

## 5. Function of hPNPase in Different Cell Compartments

### 5.1. hPNPase Controls mtRNA Trafficking

Conflicting data concerning hPNPase subcellular localization have been published over the years. Transient transfection of HO-1 cells with a construct expressing a GFP-hPNPase fusion protein, with the GFP at the N-ter, originally indicated cytoplasmic localization of hPNPase [7], but it is likely that the presence of the GFP at the N-ter, where also the mitochondrial localization signal is located, had impaired transport of the fusion protein into mitochondria. Indeed, cell fractionation and immunofluorescence studies showed that endogenous hPNPase and overexpressed C-terminal-tagged hPNPase localized (also) in mitochondria [8,85].

M.A. Teitell’s group, by using *Saccharomyces cerevisiae* mitochondria expressing hPNPase, detected hPNPase in the intermembrane space (IMS) as an inner membrane peripheral protein involved in RNA import, a result that they confirmed also in mouse liver mitochondria [16,19,86]. hPNPase import into yeast mitochondria IMS is energized by the electrochemical membrane potential and involves the i-AAA (ATPases associated with several diverse cellular activities) chaperone-protease Yme1 and the concerted activity of the translocases of the outer and inner mitochondrial membrane (TOM and TIM23, respectively) [87]. Once the N-ter MLS of hPNPase has entered the matrix through TIM23, it is cleaved by the matrix-processing peptidase MPP. The MLS is followed by a hydrophobic region (amino acids 45–51) that may act as a stop-transfer domain causing the arrest of hPNPase translocation through TIM23. Finally, Yme1 binds to the unfolded hPNPase and pulls it as it emerges from the TOM complex into the IMS where mature hPNPase assembles into a trimeric complex [87].

Translocation of hPNPase into mitochondria may be regulated by cytoplasmic proteins. It was found that in the cytosol, hPNPase forms a complex with the TCL1 factor, an oncoprotein promoting B- and T-cell malignancies in humans and transgenic mice [88]. Overexpressed TCL1 suppresses PNPase mitochondrial localization in mouse embryonic cells, impairing mitochondrial function. This phenomenon is relevant during somatic reprogramming because it switches the cell energetic metabolism from oxidative phosphorylation (OXPHOS), on which somatic cells rely, to glycolysis, which is typical of stem cells that have immature mitochondria [89,90]. A switch to glycolytic metabolism is also the base of the so-called “Warburg effect” typical of some cancer cells [91]. It remains to be established whether TCL1 retention of hPNPase in the cytoplasm occurs also in normal cells, especially in oocytes and early embryos, which are enriched for TCL1 [92,93], contributing to the regulation of mitochondrial maturation. Embryonic lethality of mice lacking PNPase and ubiquitous PNPase expression in the initial phases of zebrafish development [14,29] are consistent with the role of the protein during embryogenesis. 

**Figure 3:**
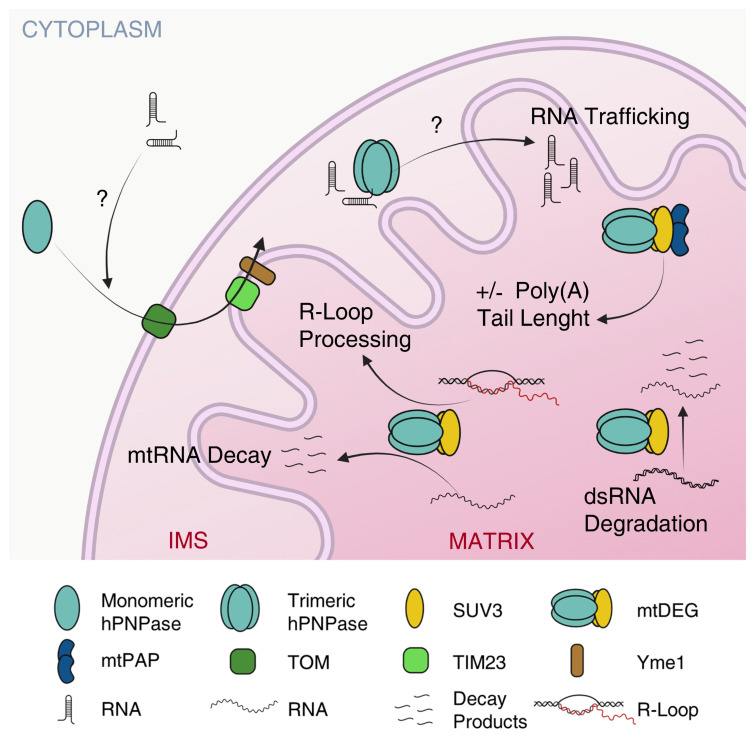
hPNPase mitochondrial functions. RNA to be imported may cross the mt outer membrane through the TOM translocon. hPNPase in the IMS facilitates RNA transport to the matrix with an unclear mechanism. In the matrix, the mtDEG regulates the decay and the polyadenylation of mtRNA, the degradation of dsRNA, and the processing of R-loops. Created with Biorender.com. Available online: https://biorender.com/ (accessed on 27 January 2022).

The main function ascribed to hPNPase in the IMS is to facilitate the translocation of non-coding RNAs from the cytosol to the mitochondrial matrix (Figure 3) [14].

Among the substrates imported by hPNPase, the *RNase P* H1 RNA, *MRP* RNA, and *5S* rRNA have been found [14]. The contribution of such RNAs (and in turn, of their import) to mitochondrial function is controversial [94]. A 20 nt long region forming a stem-loop originally identified in the *RNase P* H1 RNA promotes hPNPase-dependent translocation when ectopically fused to other RNAs, suggesting that the stem-loop is the signal recognized by hPNPase in RNAs to be imported [14,95,96]. The recognition is mediated by the S1 domain, which is required for binding the stem-loop of *MRP* RNA [58]. Recently, it was reported that the telomerase *TerC* RNA has an H1 RNA-like stem-loop and it is also imported into mitochondria. However, deletion of the stem-loop actually increases the efficiency of the *TerC* RNA translocation into mitochondria and the role of hPNPase in this pathway is unclear [96].

How hPNPase imports RNAs without degrading them and what is the exact function of hPNPase in the RNA import mechanism, which is still largely unknown in mammalian mitochondria, is unclear [97]. The *5S* rRNA and other non-coding RNAs are translocated into yeast and human mitochondria in a complex with a bound protein, which is the matrix protein rhodanese in the case of the *5S* rRNA, through a pathway presumably relying on the pre-protein translocation TOM/TIM machinery [97,98]. hPNPase stimulates the import of *5S* rRNA in both human and yeast mitochondria [14]. Further analyses are needed to understand whether hPNPase controls an additional import pathway or participates in the rhodanese-dependent one. Given the only residual levels of *5S* rRNA imported in rhodanese-deficient cells [98], the second hypothesis seems more likely.

EcPNPase can switch from an RNA degrading to an RNA carrier (and stabilizing) mode when it interacts with some small regulatory RNAs (sRNAs) in presence of the Hfq protein [41]. When EcPNPase is in the RNA carrier mode, the KH-S1 module has an “open” conformation that keeps the bound sRNA away from the core channel where the active site is located. Positively charged residues forming a loop in the S1 domain have a pivotal role in the RNA carrier complex formation [41]. hPNPase may also bind RNA substrates differently according to their intrinsic structural characteristics. Indeed, it has been suggested that the imported RNAs may be resistant to hPNPase-dependent degradation because they lack the 3′-end single-stranded tail required for the entry of the RNA molecule into the KH pore and the central channel [35]. S1 domain-dependent binding of RNAs that are not good substrates for hPNPase degradation could protect them from digestion by other nucleases and/or deliver them to a still unidentified inner membrane transporter. Noteworthy, hPNPase ectopically expressed in yeast stimulates mitochondrial RNA import [14], suggesting that the components of the import pathway interacting with hPNPase are highly conserved between yeast and humans, and/or that hPNPase has (also) a “stand-alone” function, compatible with RNA binding and protection.

It is tempting to speculate that hPNPase may have a surveillance role in RNA trafficking between the cytoplasm and the mitochondrial matrix. Surveillance by hPNPase on mtRNAs escaping from mitochondria has been documented, as hPNPase prevents the induction of an IFN auto-inflammatory response due to the release of mt-dsRNA into the cytoplasm by two mechanisms, namely degrading them, thus lowering their amount (see Section 5.2), and retaining them inside the mitochondria [73]. hPNPase may also surveil RNAs entering mitochondria, degrading or protecting them according to their structural features.

### 5.2. hPNPase Controls mtRNA Decay and Prevents mtDNA Loss

Localization of hPNPase in the IMS was unexpected because hPNPase was supposed to regulate mitochondrial RNA stability within the mitochondrial matrix, where transcription and translation take place. Consistent with an IMS localization, it was reported that hPNPase knockdown by RNA interference did not affect mtRNA levels [16]. However, successive works showed that hPNPase silencing causes the accumulation of RNA degradation intermediates and increases the stability of mtRNA, pointing to a role of hPNPase in mtRNA decay [99,100]. Indeed, it was found that a small fraction of hPNPase is located in the mitochondrial matrix [99,101], where it is part of a complex with the SUV3 helicase (also known as SUPV3L1) (Figure 3) [64,99,102]. Which portion of hPNPase directly binds SUV3 to form the complex is unknown.

SUV3 is a DExH-box RNA helicase that in vitro can unwind RNA/RNA, RNA/DNA, and DNA/DNA duplexes [64,103]. Physical association between PNPase and an RNA helicase, which allows to efficiently digest structured RNAs, has been maintained throughout evolution in distantly related organisms. In bacteria, a fraction of PNPase is associated with other proteins in a multisubunit complex, the RNA degradosome, which has a heterogeneous composition in different bacteria, modulable in response to various stresses, but that almost invariably comprises a DEAD-box RNA helicase [104,105,106,107,108,109]. In *E. coli*, the RNA helicase RhlB and PNPase, besides being subunits of the RNA degradosome, can form a 380 kDa complex at a molar ratio of 2:3, able to degrade dsRNA substrates [110]. Similarly, SUV3 and hPNPase form the mitochondrial degradosome (mtDEG), i.e., a 330 kDa heteropentameric complex at a 2:3 molar ratio able to efficiently degrade dsRNA from the 3′ to the 5′ end in presence of ATP [64]. The overexpression of a catalytically inactive SUV3, still able to bind hPNPase, inhibits mtRNA decay, whereas mtRNA degradation is not impaired if the catalytically inert SUV3 carries also another mutation preventing the interaction with hPNPase (i.e., the deletion of amino acids 510–514 [64]) [99]. The most straightforward interpretation of these data is that impaired RNA degradation is due to the formation of a defective mtDEG, in which the mutant SUV3 still able to form the complex has replaced the wt protein, thus implying a pivotal role for the mtDEG in efficient mtRNA decay [99]. Also, in *Saccaromyces cerevisiae*, which lacks a PNPase orthologue, the ScSUV3p (*S. cerevisiae* SUV3) forms a complex with the hydrolytic ribonuclease Dss1p that plays a crucial role in mtRNA metabolism [111], once again underscoring how the physical association between a helicase and a ribonuclease is beneficial to the cells and conserved throughout evolution.

An important function of the mtDEG is to limit the amount of dsRNA in mitochondria by degrading the antisense RNAs deriving from the transcription of the light strand of the mitochondrial circular genome (Figure 3) [73]. The antisense RNAs are G-rich because of the composition of mtDNA, which contains an unequal proportion of guanines between the two DNA strands [112]. hPNPase high binding affinity to poly(G) observed in vitro [59] may represent an adaptation to this peculiar feature of mtDNA that in turn determines differential composition of coding vs. antisense (non-functional) RNAs [112].

*PNPT1* depletion causes the accumulation of dsRNA of mitochondrial origin in the cytoplasm, which triggers auto-inflammation. Conversely, SUV3 depletion does not cause inflammation because the dsRNA accumulates only inside mitochondria. This suggests that hPNPase may control both the degradation of dsRNA as a subunit of the mtDEG and its retention inside the mitochondria as an isolated protein (or at least, not complexed with SUV3) [73,99,112,113,114]. Moreover, the mtDEG plays a pivotal role in the removal of R-loops, structures consisting of a displaced DNA strand and an RNA-DNA hybrid, which is processed by the mtDEG, thus contributing to mitochondrial genome stability [115].

In human cells, the mtDEG forms in distinct foci in the mitochondrial matrix, named D-foci, 50% of which co-localizes with mtRNA and/or mtDNA [99]. Like the bacterial degradosome, which has a variable composition [108], also the mtDEG can associate with different partners that modulate its activity. One of these interactors, with which the mtDEG forms a transient complex, is the mitochondrial polyadenyl polymerase (mtPAP). The complex is assembled on SUV3, which binds both hPNPase and a mtPAP dimer, and in vitro, it controls the RNA poly(A) tail lengths according to the Pi/ATP ratio, shortening them when the energy charge is low and Pi/ATP ratio is high [116]. Interestingly, the interaction between PNPase and poly(A) polymerase is evolutionarily conserved, as PAPI, the bacterial poly(A) polymerase, and PNPase form a complex in *E. coli*, which, together with the RNA degradosome, has been implied in regulating the length and deposition along mRNAs of poly(A) tails [117,118].

Pull-down experiments showed that other factors, namely LRPPRC, GRSF1, and C1QBP, are also associated with the mtDEG. In the case of GRSF1, the interaction is mediated by RNA [113], and, likely, the same applies also to LRPPRC and C1QBP, which both can bind mitochondrial mRNAs. LRPPC, in complex with SLIRP, binds to coding sequences protecting them from 3’ exonucleolytic mRNA degradation by mtDEG and promoting polyadenylation [100], whereas C1QBP binds mRNAs and promotes their translation [119]. Concerning GRSF1, it contains an RNA-binding domain, namely the quasi-RNA recognition motifs (qRRM), that specifically recognizes G-rich RNA stretches [120]. GRSF1 melts G4 RNA structures in mtRNAs facilitating their degradation by mtDEG. Notably, a GRSF1 orthologue is absent in Bacteria, where G4 sequences are rare [113]. Conversely, in human mitochondria, whose genome is enriched for G4 sequences, a substantial fraction of mtDEG-containing foci was found to co-localize with GRSF1, especially when the mtDEG was catalytically inactive, suggesting that GRSF1 may cooperate with the mtDEG to enhance its activity [113].

Besides the activity, also the formation of the mtDEG complex may be regulated. The BMI1 protein, a nuclear factor a small share of which was found to localize to the mitochondrial inner membrane (IM), forms a complex with hPNPase not including SUV3 in different mammalian cells, including human ones [121]. As the mtDEG assembles in the mitochondrial matrix [99,114], BMI1 may sequester hPNPase at the IM, thus preventing the formation of the mtDEG. Consistent with this hypothesis, BMI1 acts as a negative regulator of RNA degradation by hPNPase leading to the stabilization of different mitochondrial mRNAs [121].

### 5.3. Extra-Mitochondrial hPNPase Controls Cell Proliferation and Apoptosis

Although hPNPase is primarily a mitochondrial protein, its presence in the cytosol has been described, mainly linked to its over-expression or to severe stress conditions that may cause mitochondrial outer membrane permeabilization (MOMP) and the leakage of hPNPase into the cytosol.

hPNPase induces cell cycle arrest in G1 and apoptosis in HO-1 melanoma cells by degrading the *c-myc* mRNA. This phenomenon occurs in cells transfected with an adenoviral vector overexpressing the full length (with the MLS) protein or upon treatment with INF-β, which induces *PNPT1* expression [7,85,122,123]. Why *c-myc* mRNA should be a preferred substrate for hPNPase is not clear. The possibility that hPNPase is recruited to the *c-myc* mRNA through the interaction with an unidentified protein binding the transcript is in contrast with preferential degradation of *c-myc* mRNA over other transcripts observed in vitro, which suggests that hPNPase may recognize a peculiar structure/sequence present in the *c-myc* transcript [122]. hPNPase variants lacking both the KH and S1 RNA binding domains or either the RPH1 or the RPH2 domain are still able to degrade *c-myc* mRNA in vitro and to down-regulate the *c-myc* mRNA and induce growth arrest in melanoma cells when expressed from an adenoviral vector [85]. Although these data should be taken with some caution, as preferential *c-myc* degradation was observed in vivo upon over-expression of hPNPase variants and only partial degradation was observed in vitro after prolonged incubation of the mRNA with the proteins [85,122], they imply that recognition of *c-myc* mRNA is mediated by one of the two RPH domains. Moreover, since it is uncertain whether the unprocessed hPNPase (retaining the MLS), and even more so, the variants lacking one of the two RPH domains, can form trimers [58], *c-myc* mRNA degradation could be performed (also?) by a monomeric form. In fact, ectopic expression in the cytosol of hPNPase devoid of the MLS, which is expected to trimerize, triggers degradation of various mRNAs and apoptosis in HCT116 cells, suggesting that the activity of the processed form of hPNPase is not specific for *c-myc* mRNA [65].

hPNPase may participate in the INF-β-induced terminal differentiation of melanoma cells also through another mechanism. In fact, hPNPase down-regulates the expression of a subset of miRNAs including miR-221, which is a negative regulator of two cell proliferation controllers, namely CDKN1B/p27 and CDKN1C/p57 [124,125]. Also in this case, why hPNPase should preferentially attack miR-221 over other miRNAs is not known.

Cytosolic localization of hPNPase was described upon MOMP caused by the exposition of different types of human cells to a pro-apoptotic stimulus [16,65,126]. The protein is released from the IMS and induces massive degradation of polyadenylated RNAs and other transcripts whose 3′-ends are not protected by secondary structures, causing cell death. Silencing hPNPase expression by interference prevents mRNA degradation and reduces apoptosis in various human cells exposed to different pro-apoptotic stimuli, highlighting the relevance of hPNPase in the apoptotic pathway [65].

Interestingly, a small fraction of hPNPase seems to localize in the nuclei of cells exposed to a pro-apoptotic stimulus [65]. Moreover, two different groups [50,127] reported hPNPase nuclear localization in MDA-MB-468 cells, a highly proliferative human cell line derived from a patient with triple-negative metastatic breast cancer [128]. Two unrelated observations suggest that, at least in this cell type, the presence of hPNPase in the nucleus may be linked to proliferation and apoptosis reduction. In fact, nuclear hPNPase in MDA-MB-468 was found to physically interact with AGO1x, a translational readthrough isoform of Argonaute 1 that, like hPNPase, prevents the accumulation of endogenous dsRNA that leads to IFN response activation and enhanced apoptosis [127]. The other observation is that upon MDA-MB-468 treatment with ionic radiation, hPNPase co-immunoprecipitates with nuclear Epidermal Growth Factor Receptor (nEGFR) [50]. nEGFR activates DNA-dependent protein kinase (DNAPK), which phosphorylates hPNPase at Ser-776, a residue conserved among humans, mice, and rats, and absent from bacterial PNPases, which have a shorter S1 domain [3]. Phosphorylation of Ser776 in the S1 domain or the phosphomimetic point mutation Ser776Glu abolishes in vitro degradation of *c-myc* mRNA by hPNPase [50], increasing radioresistance and reducing apoptosis. Considering the position of the phosphorylated residue, it is possible that the RNA binding activity of hPNPase may be affected by phosphorylation. It remains to be established whether the inhibitory effect of phosphorylation on hPNPase degradative activity is specific to *c-myc* mRNA or degradation of other RNAs is also impaired.

## 6. Conclusions

Our understanding of the physiological role of hPNPase as it emerges from this review is still fragmentary, the studies on hPNPase being limited in number and performed on different cell types and conditions. Further investigations are undoubtedly needed to get a more exhaustive picture of the many functions played by this ancient protein in human cells.

We have a partial understanding of how hPNPase activities observed in vitro, in biochemical assays, translate into activity in the cells. How RNAs with different structural features are recognized and bound by hPNPase, which ultimately determines whether they will be degraded or not, is unclear. Answering this question is of pivotal importance to understand the role played by hPNPase in RNA import into mitochondria, its preference for some RNAs, like *c-myc* or mir-221, and also for oxidized RNA. This is an evolutionarily conserved feature of PNPase, as both EcPNPase and hPNPase bind, but do not degrade, oxidized RNAs containing 8-oxo-7,8-dihydroguanine (8-oxoGua) and in both organisms, PNPase is involved in the response to oxidative stress [129,130,131]. Another completely overlooked aspect is the in vivo relevance of the NDPs polymerizing activity of hPNPase observed in vitro. As tails added by hPNPase should be heteropolymeric, in principle they could be identified by mtRNA sequencing and distinguished from the poly(A) tails added by the mtPAP.

A very relevant aspect that needs clarification is how different stimuli and genetic backgrounds impact and modulate hPNPase intracellular (and intra-mitochondrial, between the IMS and the matrix) trafficking. Given the role played by hPNPase in proliferation and apoptosis control, understanding the mechanisms controlling hPNPase trafficking among cell compartments may have important implications for cancer research.
